# Association between Diet Quality and Adiposity in the Atlantic PATH Cohort

**DOI:** 10.3390/nu9101155

**Published:** 2017-10-21

**Authors:** Vanessa DeClercq, Yunsong Cui, Cynthia Forbes, Scott A. Grandy, Melanie Keats, Louise Parker, Ellen Sweeney, Zhijie Michael Yu, Trevor J.B. Dummer

**Affiliations:** 1 Population Cancer Research Program, Department of Pediatrics, Dalhousie University, Halifax, NS B3H 4R2, Canada; yunsong.cui@dal.ca (Y.C.); cynthia.forbes@dal.ca (C.F.); louise.parker@dal.ca (L.P.); ellen.sweeney@dal.ca (E.S.); zhijie.m.yu@gmail.com (Z.M.Y.); 2 School of Health and Human Performance, Dalhousie University, Halifax, NS B3H 1T8, Canada; scott.grandy@dal.ca (S.A.G.); melanie.keats@dal.ca (M.K.); 3 Centre of Excellence in Cancer Prevention, School of Population and Public Health, University of British Columbia, Vancouver, BC V6T 1Z3, Canada; tdummer@mail.ubc.ca

**Keywords:** diet quality, body mass index, abdominal obesity, fat mass

## Abstract

The aim of this study was to examine diet quality among participants in the Atlantic Partnership for Tomorrow’s Health (PATH) cohort and to assess the association with adiposity. Data were collected from participants (*n* = 23,768) aged 35–69 years that were residents of the Atlantic Canadian provinces. Both measured and self-reported data were used to examine adiposity (including body mass index (BMI), abdominal obesity, waist-to-hip ratio and fat mass) and food frequency questionnaires were used to assess diet quality. Overall, diet quality was statistically different among provinces. Of concern, participants across all the provinces reported consuming only 1–2 servings of vegetables and 1–2 servings fruit per day. However, participants also reported some healthy dietary choices such as consuming more servings of whole grains than refined grains, and eating at fast food restaurants ≤1 per month. Significant differences in BMI, body weight, percentage body fat, and fat mass index were also observed among provinces. Adiposity measures were positively associated with consumption of meat/poultry, fish, snack food, sweeteners, diet soft drinks, and frequenting fast food restaurants, and inversely associated with consumption of whole grains and green tea. Although all four provinces are in the Atlantic region, diet quality vary greatly among provinces and are associated with adiposity.

## 1. Introduction

Life expectancy is lower [1] and the prevalences of most chronic conditions are much higher in the Atlantic region of Canada, including Newfoundland and Labrador, New Brunswick, Nova Scotia, and Prince Edward Island [2,3]. Suboptimal dietary habits have been associated with mortality due to chronic disease [4]. A “Prudent”-type diet (high intake of vegetables, fruits, fish, poultry, whole grains, and low-fat dairy products) has been associated with a lower risk of mortality compared to a “Western”-type diet (characterized by high intake of meat, processed meat, bread, dairy products, coffee, black tea, soft drinks, dressing, sauce, and mayonnaise) [5,6].

Poorer diet quality has been shown to increase the risk of many chronic conditions [7,8,9]. Studying overall diet quality rather than isolated nutrients to characterize a population’s dietary intake has gained wide acceptance in nutritional epidemiology. Diet quality can be assessed by several different indices, with lower scores (i.e., less healthy diets) associated with significantly higher incidences of cancer, diabetes, cardiovascular disease, and mortality [9]. Furthermore, with a well-established link between these conditions and obesity, there is a need to focus efforts on dealing with the global rise in obesity [10]. 

Many variables contribute to obesity including environmental, genetic, and lifestyle factors such as sleep duration, physical inactivity and poor diet [11,12]. A recent systematic review by Asghari et al. reported an inverse association between body mass index (BMI) and diet quality, as assessed by the healthy eating index (HEI) [13]. The HEI and its newer revised versions are methods to assess diet quality based on the Dietary Guidelines for Americans [14] and have been adapted to correspond with Canada’s Food Guide [15,16]. The Atlantic region of Canada is an area of the country with particularly high rates of obesity [17]; however, there is very little nutritional epidemiological research examining region-specific diet quality in the area. Therefore, the aim of this study was to assess diet quality among Atlantic Canadian provinces and explore the association with obesity in participants of the Atlantic Partnership for Tomorrow’s Health (PATH) cohort.

## 2. Materials and Methods

### 2.1. Study Population and Data Collection

Data for the Atlantic PATH cohort were collected during 2009–2015 from the four Atlantic Canada provinces: New Brunswick, Newfoundland and Labrador, Nova Scotia, and Prince Edward Island. Dietary questionnaire data were collected from 23,768 participants aged 35–69 years. Of these participants, 19,393 also had anthropometric measures required to calculate BMI. Anthropometric measures, including height, weight, and waist and hip circumference, were assessed by research nurses in assessment centers and self-reported by participants as part of the questionnaire data.

### 2.2. Assessment of Demographic and Lifestyle Risk Factors

Demographic and lifestyle data including sex, age, education, smoking, alcohol consumption and physical activity were assessed. The level of education completed was categorized as high school or less, college level, and university level or higher. For smoking behavior, participants were categorized as current smoker, former smoker, and never smoked. For alcohol consumption, participants were classified as an abstainer, occasional drinker (<3 times/month), regular drinker (1–3 times/week), and habitual drinker (4–7 times/week). Binge drinking of alcohol was reported when ≥5 and ≥4 drinks were consumed on the same occasion for men and women, respectively. Participants were categorized into never binge drinking, <1 per month, 1–3 times per month, once a week, or >1 per week. Levels of physical activity were collected using open-ended questions in the International Physical Activity Questionnaire (IPAQ long form) [18]. Participants were asked to identify the frequency and duration of all vigorous, moderate, and walking physical activity (i.e., inclusive of occupational, domestic and leisure-time) within the last seven days. For all domains, metabolic equivalents of a task (MET) values were calculated and summed to obtain a total physical activity score in MET-minutes/week. In compliance with the IPAQ scoring protocol [19], categorical physical activity scores were defined as inactive, moderately active, and active.

### 2.3. Assessment of Obesity and Body Composition

To classify participants as obese/not obese, measured anthropometric data were used to calculate BMI and waist-to-hip ratio, and when anthropometric indices were not available, self-reported measures were used. BMI was calculated by dividing body weight by height in meters squared. Participants with a BMI of <18.5, 18.5–24.9, 25.0–29.9 and ≥30.0 kg/m^2^ were considered underweight, normal weight, overweight and obese, respectively [20]. The waist-to-hip ratio was set at >0.90 for men and >0.85 for women [21]. Abdominal obesity was defined as having a waist circumference ≥102 cm for men and ≥88 cm for women [21,22]. Bioelectrical impedance (Tanita Segmental Body Composition Analyzer) was used to assess body composition for participants who visited an assessment center. Fat mass and fat-free mass indices were calculated by dividing fat mass and fat-free mass by height in meters squared, respectively.

### 2.4. Assessment of Diet Quality

Dietary assessments of participants were collected via the food frequency questionnaire at the time of enrollment in the study. The food listed in the questionnaire were categorized into six major groups: (1) fruits and vegetables; (2) dairy products; (3) grains; (4) meats and alternatives; (5) desserts and snacks; and (6) beverages and miscellaneous. Each participant was required to recall food intake over the past 12 months, indicating the frequency with which they usually consumed each item, choosing rarely/never, servings per day, servings per week, or servings per month. Additionally, participants were requested to indicate the number of servings habitually consumed in a typical day for fruits and vegetables, dairy products, grains, meat and alternatives. The food frequency questions were used to calculate a HEI score for each participant, producing a continuous variable with a minimum score of 0 (indicative of poorest dietary habits) and a maximum score of 60 (indicative of optimal dietary habits), as previously described [22].

### 2.5. Statistical Analysis

Data analyses were performed with IBM SPSS Statistics software (version 22, SPSS Inc., Chicago, IL, USA) and a *p*-value of <0.05 was considered statically significant. Chi-square analyses were used to determine significant associations between provinces and demographic (sex, age, and education), behavioral (physical activity, drinking, and smoking), and adiposity measures. Categorical variables were presented as counts (%) and continuous variables were presented as mean ± standard deviation (SD). Differences in continuous anthropometric data among provinces were analyzed using ANOVA, if necessary followed by post hoc two-tailed *t* tests adjusted with a Bonferroni correction for multiple comparisons. Spearman’s correlation coefficients were used to assess the relationship between BMI and dietary habits. Log-binomial regression models were used to evaluate dietary variables associated with obesity. The association between obesity and each dietary variable are presented as prevalence ratios with 95% confidence intervals (95% CI). For the development of the statistical models for predicting the probability of having a BMI > 30, both demographic variables and lifestyle behaviors were included in the regression models. Variables with a *p* < 0.05 (sex, age, education, smoking, alcohol use, physical activity, and province) were retained in the final regression models.

## 3. Results

### 3.1. Adiposity, Demographic and Lifestyle Characteristics

There were more female than male participants overall, however, there was no statistical difference in sex distribution among the provinces. Most participants were 50–59 years of age. Overall, nearly half (46%) of participants have university level education, with a lower percentage in Newfoundland and Labrador (37%), and Prince Edward Island (38%). The lifestyle behaviors of smoking, alcohol drinking, and physical activity also statistically differed among provinces. Newfoundland and Labrador (13%), and Prince Edward Island (11%) reported the highest percentage of current smokers, Nova Scotia reported the highest habitual drinking (18%), and Prince Edward Island reported the highest percent of inactive participants (18%) (Table 1). 

Statistically significant differences in multiple measures of adiposity were also observed among provinces (Table 1). Newfoundland and Labrador, Prince Edward Island, New Brunswick, and Nova Scotia consistently reported highest to lowest for BMI, body weight, percentage body fat, and the fat mass index. This was reflected by Newfoundland and Labrador having the highest, and Nova Scotia having the lowest proportion of participants with a BMI in the obese range (≥30 kg/m^2^) (59% vs. 24%), abdominal obesity (57% vs. 49%) and a waist-to-hip ratio above guidelines (68% vs. 62%) (Table 1). 

### 3.2. Diet Quality

The average HEI score was 39.1 for the whole region and this was statistically different among provinces with Newfoundland and Labrador receiving the lowest score (37.8) and New Brunswick receiving the highest score (39.8) (Table 1). Fruit and vegetable intake were statistically different among provinces (Table 2). Overall, most participants reported consuming five or more servings of fruits and vegetables on five or more days per week, with the highest percentage in Nova Scotia (52%) and the lowest in Newfoundland and Labrador (38%). However, most participants only reported consuming 1–2 servings of vegetables (53%) and 1–2 servings of fruit per day (59%) (Table 2). Newfoundland and Labrador reported the lowest percentage of individuals who consumed five or more servings of fruit and vegetables per day (50% in Newfoundland versus 61% in other provinces). 

The intake of grains, milk and dairy, and meat and alternatives varied statistically among provinces. In general, most participants (62%) reported consuming <2 servings of whole grains and 54% reported consuming <2 servings of refined grains per day (Table 2). Most participants (68%) reported consuming two or more servings of milk per day and skim milk was the most commonly reported type of milk consumed (Table 2). Overall, the majority of participants reported one serving of meat/poultry per day, and New Brunswick and Nova Scotia had the highest percentage of participants (12%) consuming three or more servings per day (Table 2). Nova Scotia also had the highest percentage of participants (44%) consuming three or more servings of eggs per day (Table 2). Most participants (91%) reported either none or one serving of fish per day, with Newfoundland and Nova Scotia more likely to consume one serving per day (Table 2). Tofu and bean curd were not commonly consumed among participants (<6% consuming daily), with the lowest rates reported in participants from Newfoundland and Labrador, and Prince Edward Island, and highest in participants from Nova Scotia and New Brunswick (Table 2). Regionally, most participants did not consume beans or legumes daily. Prince Edward Island reported the lowest servings (36% consumed daily) and Nova Scotia and New Brunswick the highest (45% consumed daily) (Table 2). In total, most participants (64%) consumed at least one serving of nuts or seeds per day, except in Newfoundland and Labrador where daily consumption was much less (53%) (Table 2). 

Beverage (soft drink, coffee, and tea) consumption and frequency of garlic, hot spices, and ginger were statistically different among the four provinces (Table 3). Daily soft drink consumption was most prevalent whereas daily coffee consumption was less common in Newfoundland and Labrador. Consumption of garlic, hot spices, and ginger on more than five days per week was most common in Nova Scotia. Forty-five percent and 34% of participants reported consuming snack foods and desserts 1–2 times per week, respectively, whereas 36% of participants reported never using salt and 47% reported visiting fast food restaurants ≤1 per month (Table 3). Overall, the majority (60%) of participants used a combination of two or more fats/oil, with olive oil and canola being the most common combination, and margarine being most commonly used on bread (Table 3). 

### 3.3. Relationship between Adiposity and Dietary Habits

Servings of meat/poultry, refined grains, coffee, snack food, sweetener use, soft drinks, and frequency of eating at fast food restaurants were all positively associated with BMI. In contrast, the frequency of garlic, hot spices, and ginger; five or more serving of fruit and vegetables; whole grains; tofu; nut/seeds; green tea; green vegetables; all vegetables; fruit, juice; and the HEI were all inversely associated with BMI (Table 4). Waist circumference and percentage body fat were also positively associated with meat/poultry, fish, snack foods, sweeteners, diet soft drinks, and fast-food, and negatively with whole grains (Table 4). 

Since there were clear differences in diet quality and adiposity measures among provinces, regression models were used to identify dietary variables that were predictive of obesity (or an obese BMI). Several dietary factors were found to be statistically associated with an obese BMI. In the unadjusted model, participants consuming higher servings of meat/poultry and refined grains were more likely to be obese (Table 5). By contrast, participants were less likely to be obese with increased servings of green vegetables, whole grains, tofu, nuts/seeds, vegetables and fruits (Table 5). Participants with a higher score on the HEI were less likely to be obese (Table 5). All relationships, with the exception of green vegetables, remained statistically significant after adjusting for additional co-variables (Table 5). By controlling for the co-variables, the regression models enabled us to test the strength of the associations between the exposure variables (diet quality) and study outcomes (obesity), and the chi-square goodness of fit test indicated that the model was a good fit (*p* > 0.05).

## 4. Discussion

Suboptimal diet quality has emerged as an important contributor to chronic disease [4,5,6,7,8,9]. In this study, we compared diet quality of adults in four Atlantic Canadian provinces and assessed the association between diet quality and obesity. Based on the regression models, participants were more likely to be obese if they consumed more meat/poultry, refined grains, soft drinks, snack foods, sweeteners, and visited fast food restaurants, and were less likely to be obese if they more frequently consumed garlic, hot spices and ginger, fruit and vegetables, whole grains, milk products, tofu, and nuts/seeds. Although these associations are not surprising, the differences in diet quality across the regions, as well as differences in measures of adiposity among provinces, highlights the need for distinct health policies and programs in each province to help combat the rising concern of obesity and related co-morbidities.

Regional studies have identified individual food trends and relationships between specific food items and disease. For example, there have been reported differences in sodium intake among people living in different areas of the United States [23] and, similar to the current study, a significant inverse relationship was observed between measures of adiposity (BMI and waist circumference) and whole grain consumption [24]. However, rather than focusing on individual food items, there has recently been a shift to exploring overall diet quality and the risk of disease or mortality [4,5,6,7,8,9]. 

Based on patterns of food intake, the American Heart Association has made recommendations for optimal cardiovascular health that focus on a combination of dietary components such as fruit and vegetables, fish, sodium, sugar-sweetened beverages, whole grains, nuts, seed and legumes, processed meat and saturated fat [25]. More specifically, excess sodium intake, processed meats, sugar sweetened beverages, and low intake of nuts/seeds, seafood omega-3 fats, vegetables, fruits and whole grains are associated with cardiometabolic deaths [4]. Importantly, the findings varied by sex, age, race, and education but, unfortunately, there were no region-specific data included. 

Chen et al. identified four main dietary patterns in adults in Newfoundland and Labrador and showed that males were more likely to consume higher amounts of meat and fish whereas older participants were less likely to choose meat and more likely to choose fish [26]. Diet quality in the above study may be reflective of the history and culture of that specific coastal region which has traditionally been dependent on a fishing industry. Region-specific dietary data across a country are important for understanding factors that influence diet quality and developing policy, guidelines, and education programs to target those specific populations. When considering region-specific diet quality, it is also important to consider availability, particularly in rural/remote areas, and socio-demographic factors that influence diet quality. For example, household size and income influence food purchasing [27] and higher income and education levels are associated with higher fruit and vegetable consumption [28,29,30]. Furthermore, increased density of neighborhood fast-food restaurants was associated with unhealthy lifestyles such as low consumption of fruit and vegetables and not meeting physical activity guidelines [31]. Specifically, in Nova Scotia, the density of fast food restaurants has been associated with community-level material deprivation (social and/or material disadvantage) [32], an important factor that has been associated with larger waist circumference, waist-to-hip ratio, and BMI [33]. 

Few studies have investigated multiple measures of adiposity (BMI, waist circumference, waist-to-hip ratio, percent body fat) in relation to diet quality. Recently, the New Zealand Adult Nutrition Survey was used to examine the association between more than one adiposity measure (BMI and waist circumference) and diet quality [34]. In that study, an inverse association was reported between both BMI and waist circumference, and a “healthy” dietary pattern (characterized by high intake of breakfast cereal, low fat milk, soy and rice milk, soup and stock, yoghurt, fruit and tea, and low intakes of pies and pastries, potato chips, white bread, takeaway foods, soft drinks, beer and wine) compared to a “traditional” dietary pattern (characterized by high intake of beef, starchy vegetables, green vegetables, carrots, tomatoes, savory sauces, regular milk, cream, sugar, tea and coffee, and low intake of takeaway foods) [34]. A study conducted in Quebec, Canada demonstrated that participants within the highest Western-type diet tertile had the highest BMI, waist circumference, and fat mass; conversely, those in the highest Prudent-diet tertile had the lowest BMI, waist circumference, and fat mass [35]. In a national Canadian survey, higher diet quality was associated with a lower BMI and less obesity [36,37], however, it is known that access to different food sources varies [38] and consumption patterns are not equal across Canada [39]. The current study examined the distinct region of Atlantic Canada, which is known to have elevated levels of obesity and other chronic disease [2]. Findings demonstrate that those consuming low levels of fruits and vegetables, whole grains, nuts/seeds and higher levels of refined grains, meat/poultry, snack foods, sweeteners, soft drinks, and fast food were more likely to be obese. 

The current study is unique in that it looks at diet quality in a specific region of the country and utilizes multiple measures of adiposity to explore relationships with dietary components. A major strength of the current study is that both measured and self-reported physical indices were used. Additionally, this study included participants from across the Atlantic region, including both rural and urban centers. However, the current study is not without limitations. For example, the dietary information was self-reported and hence subject to bias when participants are required to recall past dietary habits. Additionally, the current study utilizes an older method for calculating diet quality, however this method was chosen for comparison and consistency with earlier data analysis conducted on this cohort and the timeline of Atlantic PATH recruitment [22]. There are newer methods for calculating the HEI for the Canadian population [40], which should be considered for future studies. Finally, the study was cross-sectional in design, thus it can only explain associations, not causal effects. However, with the longitudinal design of the Atlantic PATH study, this type of analysis will be possible in the future with follow-up assessments in these participants.

Future research should assess additional factors that may influence diet quality in a specific region, such as access and availability. Furthermore, due to geographic isolation and the importance of particular industries, it may be may be more relevant for certain areas to utilize specific dietary assessment tools [26]. Dietary questionnaires should also be expanded to include certain food items specific to the Canadian population which may positively or negatively influence obesity such as processed meats, cheese, gravies, sauces, and yogurt. 

The study information will be useful for developing policy and strategies including food pricing, availability, and marketing strategies on food selection. It could also influence food guidelines and policies or programs to address needs of specific groups or populations. Public health policies in these regions need to limit the impact of detrimental food/nutrition environments and maximize access to healthy/positive food choices. 

## 5. Conclusions

In conclusion, diet quality of participants living in the Atlantic region of Canada varied among provinces and were associated with multiple indices of obesity. These results should be used to help guide public health strategies aimed at altering dietary habits to improve health and reduce chronic disease.

## Figures and Tables

**Table 1:** Demographic and behavioral characteristics of Atlantic PATH participants.

	Total	New Brunswick	Newfoundland and Labrador	Nova Scotia	Prince Edward Island	*p* Value
	*n* (%)					
Sex						0.273
Males	6022 (31.1)	1390 (30.4)	813 (31.2)	3698 (31.2)	103 (27.2)	
Females	13,371 (68.9)	3181 (69.6)	1716 (67.9)	8164 (38.8)	275 (72.8)	
Age						<0.001
35–39	1902 (9.8)	579 (12.7)	285 (11.3)	1000 (8.4)	38 (10.1)	
40–49	5212 (27.0)	1434 (31.4)	845 (33.4)	2845 (24.0)	88 (23.3)	
50–59	6979 (36.1)	1672 (36.6)	887 (35.1)	4276 (36.1)	144 (38.1)	
60–69	5246 (27.1)	886 (19.4)	512 (20.2)	3740 (31.5)	108 (28.6)	
Education						<0.001
High school or less	3629 (18.8)	886 (19.4)	455 (18.1)	2216 (18.8)	65 (17.3)	
College level	6856 (35.5)	1600 (35.1)	1126 (44.8)	3942 (33.4)	168 (44.7)	
University level	8835 (45.7)	2078 (45.5)	933 (37.1)	5655 (47.9)	143 (38.0)	
Smoking status						<0.001
Never	9820 (51.1)	2444 (54.1)	1184 (47.3)	5994 (50.9)	171 (45.7)	
Former	7532 (39.2)	1615 (35.7)	1005 (40.1)	4732 (40.2)	161 (43.0)	
Current	1881 (9.8)	461 (10.2)	315 (12.6)	1057 (9.0)	42 (11.2)	
Alcohol drinking						<0.001
Abstainer	2121 (11.2)	524 (11.6)	417 (16.6)	1134 (9.8)	43 (11.5)	
Occasional drinker	7974 (42.0)	1964 (43.3)	995 (39.7)	9835 (41.9)	155 (41.6)	
Regular drinking	5847 (30.8)	1432 (31.6)	802 (32.0)	3486 (30.2)	113 (30.3)	
Habitual drinker	3057 (16.1)	611 (13.5)	291 (11.6)	2082 (18.0)	62 (16.6)	
Binge drinking						<0.001
Never	2440 (60.5)	1256 (59.7)	7708 (72.1)	213 (63.8)	11641 (67.6)	
< Once per month	1575 (39.1)	840 (39.9)	2204 (20.6)	115 (34.4)	4758 (27.6)	
1–3 times per month	-	-	433 (4.0)	-	438 (2.5)	
Once per week	-	-	188 (1.8)	-	188 (1.1	
>Once per week	15 (0.4)	9 (0.4)	159 (1.5)	5 (1.5)	188 (1.1)	
Physical activity						<0.001
Inactive	2340 (12.3)	612 (13.7)	358 (14.6)	1298 (11.1)	66 (18.0)	
Moderate active	5206 (27.3)	1319 (29.5)	623 (25.4)	3138 (26.7)	109 (29.7)	
Active	11,541 (60.5)	2547 (56.9)	1471 (60.0)	7302 (62.2)	192 (52.3)	
Body mass index (BMI) ≥30	5685 (29.3)	1220 (26.7)	1494 (59.1)	2815 (23.7)	142 (37.6)	<0.001
Waist-to-hip ratio *	12403 (64.7)	3100 (68.3)	1711 (68.4)	7304 (62.3)	250 (67.9)	<0.001
Abdominal obesity ^†^	9930 (51.7)	2500 (55.0)	1419 (56.7)	5780 (49.3)	201 (54.5)	<0.001
	Mean (SD)					
Height, cm	166.8 (9.7)	166.9 (9.3)	166.5 (9.1)	166.9 (9.0)	166.5 (9.8)	0.285
Weight, km	77.0 (16.5)	77.3 (15.6) ^b^	83.8 (15.2) ^a^	75.4 (16.8) ^c^	79.4 (14.8) ^b^	<0.001
Waist, cm	93.5 (15.6)	94.3 (15.5) ^a^	94.6 (16.3) ^a^	92.9 (15.4) ^b^	94.1 (14.9) ^a,b^	<0.001
Hips, cm	105.0 (12.9)	105.3 (13.3) ^a,b^	105.7 (13.2) ^a^	107.8 (12.7) ^b^	105.4 (11.3) ^a,b^	0.004
BMI, kg/m^2^	27.6 (5.2)	27.7 (4.8) ^c^	30.2 (4.7) ^a^	27.0 (5.3) ^d^	28.6 (4.7) ^b^	<0.001
Waist-to-hip ratio	0.89 (0.10)	0.90 (0.11) ^a^	0.90 (0.12) ^a^	0.89 (0.10) ^b^	0.89 (0.10) ^a,b^	<0.001
Percentage body fat, %	32.5 (8.5)	32.3 (7.8) ^b^	37.8 (7.1) ^a^	31.8 (8.6) ^b^	33.6 (8.6) ^b^	<0.001
Fat mass index, kg/m^2^	9.1 (5.9)	8.7 (2.9) ^b^	11.8 (2.8) ^a^	8.8 (6.6) ^b^	9.4 (3.6) ^b^	<0.001
Fat mass, kg	25.0 (13.9)	24.2 (7.0) ^b^	32.6 (6.3) ^a^	24.2 (15.6) ^b^	26.5 (7.1) ^b^	<0.001
Healthy eating index (HEI)	39.1 (8.6)	39.8 (8.0) ^a^	37.8 (8.9) ^d^	39.0 (8.7) ^c^	39.5 (7.6) ^a,b^	<0.001

* Waist to hip ratio ≥ 0.9 for men and ≥0.85 for women. ^†^ Waist circumference >102 cm for men and >88 cm for women. - Data suppressed due to small cell counts. For continuous variables, values in the same row not sharing the same subscript letter are statistical different (*p* < 0.05). SD, standard deviation.

**Table 2:** Diet quality for fruit and vegetables, grains, milk, and meat and alternatives.

	Total	New Brunswick	Newfoundland and Labrador	Nova Scotia	Prince Edward Island	*p* Value
	*n* (%)					
5 or more serving fruits and vegetables frequency						<0.001
0 days per week	1898 (9.8)	525 (11.5)	285 (11.3)	1033 (8.7)	47 (12.7)	
1–2 days per week	3350 (17.3)	831 (18.2)	607 (24.0)	1817 (15.3)	85 (22.5)	
3–4 days per week	4692 (24.2)	1036 (22.7)	681 (26.9)	2880 (24.3)	82 (21.7)	
>5 days per week	9444 (48.7)	2175 (47.6)	956 (37.8)	6127 (51.7)	164 (43.4)	
Fruit/vegetable juice frequency						0.084
Never	2844 (18.5)	681 (18.2)	341 (16.8)	1746 (18.9)	67 (21.9)	
1–3 times per month	5564 (36.3)	1530 (40.8)	724 (35.6)	3177 (34.5)	118 (38.6)	
1–4 times per week	3428 (22.3)	674 (18.0)	392 (19.3)	2307 (25.0)	45 (14.7)	
>5 times per week	3508 (22.9)	861 (23.0)	574 (28.3)	1986 (21.5)	76 (24.8)	
Green vegetable servings						<0.001
0 per day	1610 (14.2)	463 (10.9)	322 (14.4)	775 (17.4)	42 (12.3)	
<2 per day	8815 (77.8)	3362 (79.1)	1768 (78.9)	3386 (76.1)	268 (78.4)	
2–5 per day	759 (6.7)	354 (8.3)	130 (5.8)	243 (5.5)	28 (8.2)	
>5 per day	142 (1.3)	71 (1.7)	22 (1.0)	44 (1.0)	-	
Servings of vegetables						<0.001
0 per day	749 (3.9)	101 (2.3)	131 (5.3)	502 (4.3)	10 (2.7)	
1–2 per day	10,117 (53.1)	2411 (53.7)	1560 (63.7)	5920 (50.6)	195 (52.6)	
3–4 per day	6506 (34.1)	1542 (34.4)	637 (26.0)	4182 (35.7)	131 (35.3)	
>5 per day	1687 (8.9)	433 (9.7)	123 (5.0)	1094 (9.4)	35 (9.4)	
Servings of fruit						<0.001
0 per day	1647 (8.6)	305 (6.8)	203 (8.3)	1106 (9.5)	24 (6.5)	
1–2 per day	11,241 (59.0)	2588 (57.7)	1552 (63.3)	6841 (58.5)	230 (62.0)	
3–4 per day	5240 (27.5)	1324 (29.5)	592 (24.2)	3204 (27.4)	111 (29.9)	
>5 per day	933 (4.9)	271 (6.0)	104 (4.2)	548 (4.7)	6 (1.6)	
Servings of fruit/vegetable juice						<0.001
0 per day	9603 (50.4)	2315 (51.6)	1152 (47.0)	5930 (50.7)	183 (49.3)	
1–2 per day	8654 (45.4)	1999 (44.5)	1196 (48.8)	5256 (44.9)	176 (47.4)	
3–4 per day	667 (3.5)	145 (3.2)	85 (3.5)	425 (3.6)	10 (2.7)	
>5 per day	137 (0.7)	29 (0.6)	18 (7.0)	88 (0.8)	-	
5 or more serving fruit and vegetables per day						<0.001
No	7622 (40.0)	1740 (38.8)	1209 (49.3)	4505 (38.5)	144 (38.8)	
Yes	11,425 (60.0)	2746 (61.2)	1242 (50.7)	782 (61.5)	227 (61.2)	
Servings of whole grains						<0.001
0 per day	1224 (7.2)	382 (8.7)	290 (12.3)	517 (5.2)	32 (8.8)	
<2 per day	10,479 (61.6)	2780 (63.6)	1542 (65.3)	5890 (59.6)	233 (64.6)	
4–5 per day	4467 (26.2)	1016 (23.2)	463 (19.6)	2897 (29.3)	84 (23.2)	
>5 per day	854 (5.0)	193 (4.4)	65 (2.8)	583 (5.9)	13 (3.6)	
Servings of refined grains						<0.001
0 per day	6275 (38.1)	1523 (36.9)	691 (30.6)	3910 (40.4)	130 (37.8)	
<2 per day	8903 (54.1)	2238 (54.2)	1355 (60.1)	5100 (52.6)	186 (54.1)	
4–5 per day	1087 (6.6)	319 (7.7)	171 (7.6)	570 (5.9)	25 (7.3)	
>5 per day	197 (1.2)	46 (1.1)	38 (1.7)	110 (1.1)	-	
Servings of milk and dairy						<0.001
0 per day	687 (3.6)	161 (3.6)	88 (3.6)	419 (3.6)	17 (4.6)	
1 per day	5323 (28.2)	1179 (26.3)	714 (29.2)	3311 (28.7)	96 (26.0)	
2 per day	6910 (36.6)	1711 (38.2)	947 (38.7)	4108 (35.7)	127 (34.4)	
≥3 per day	5952 (31.5)	1431 (31.9)	697 (28.5)	3685 (32.0)	129 (35.0)	
Type of milk usually consumed						<0.001
Homogenized	592 (3.3)	135 (3.1)	114 (4.9)	330 (3.0)	13 (3.8)	
2% cow’s milk	3714 (20.6)	1100 (25.6)	524 (22.3)	1991 (18.2)	89 (26.2)	
1% cow’s milk	5934 (33.0)	1363 (31.7)	731 (31.1)	3707 (33.8)	115 (33.8)	
Skim cow’s milk	6326 (35.1)	1354 (31.5)	823 (35.0)	4034 (36.8)	97 (28.5)	
Soy	597 (3.3)	155 (3.6)	48 (2.0)	385 (3.5)	9 (2.6)	
Other	835 (4.6)	188 (4.4)	109 (4.6)	518 (4.7)	17 (5.0)	
Meat/poultry servings						<0.001
0 per day	650 (3.4)	119 (2.7)	42 (1.7)	478 (4.1)	9 (2.5)	
1 per day	9891 (52.4)	2195 (49.1)	1300 (53.1)	6187 (53.6)	182 (49.6)	
2 per day	6083 (32.2)	1604 (35.9)	826 (33.7)	3494 (30.3)	144 (39.2)	
≥3 per day	2252 (11.9)	550 (12.3)	282 (11.5)	1382 (12.0)	32 (8.7)	
Egg servings						<0.001
0 per day	2102 (11.9)	725 (17.8)	364 (16.4)	958 (8.7)	44 (12.9)	
1 per day	4352 (24.6)	1343 (33.0)	854 (38.4)	2022 (18.4)	119 (35.0)	
2 per day	4805 (27.2)	985 (24.2)	494 (22.2)	3236 (29.4)	80 (23.5)	
≥3 per day	6428 (36.3)	1013 (24.9)	513 (23.1)	4792 (43.5)	97 (28.5)	
Fish servings						0.005
0 per day	7455 (46.9)	1889 (49.0)	909 (44.3)	4482 (46.6)	149 (49.2)	
1 per day	7172 (45.2)	1671 (43.4)	947 (46.2)	4408 (45.8)	131 (43.2)	
2 per day	1015 (6.4)	242 (6.3)	157 (7.7)	590 (6.1)	23 (7.6)	
≥3 per day	237 (1.5)	50 (1.3)	38 (1.9)	148 (1.5)	-	
Tofu or bean curd servings						<0.001
0 per day	12,425 (95.1)	3516 (94.7)	1873 (97.9)	6515 (95.0)	287 (98.0)	
1 per day	563 (4.3)	205 (5.5)	35 (1.8)	316 (4.5)	6 (2.0)	
2 per day	44 (0.3)	15 (0.4)	-	26 (0.4)	-	
≥3 per day	18 (0.1)	-	-	12 (0.2)	-	
Bean or other legume servings						<0.001
0 per day	8645 (57.5)	2308 (59.6)	1235 (55.5)	4889 (55.5)	188 (63.6)	
1 per day	5718 (38.0)	1388 (35.8)	695 (34.6)	3512 (39.9)	110 (35.9)	
2 per day	551 (3.7)	145 (3.7)	65 (3.2)	334 (3.8)	6 (2.0)	
≥3 per day	123 (0.8)	34 (0.9)	13 (0.6)	74 (0.8)	-	
Nut or seed servings						<0.001
0 per day	5842 (36.2)	1411 (34.7)	980 (47.4)	3324 (34.5)	111 (34.9)	
1 per day	8464 (52.5)	2191 (54.0)	913 (44.2)	5159 (53.5)	182 (57.2)	
2 per day	1492 (9.3)	382 (9.4)	145 (7.0)	940 (9.8)	22 (6.9)	
≥3 per day	329 (2.0)	77 (1.9)	28 (1.4)	218 (2.3)	-	

- Data suppressed due to small cell counts.

**Table 3:** Diet quality for beverages and miscellaneous intake.

	Total	New Brunswick	Newfoundland and Labrador	Nova Scotia	Prince Edward Island	*p* Value
	*n* (%)					
Most often eaten on bread						<0.001
Butter	5706 (29.9)	1360 (30.2)	411 (16.6)	3768 (32.3)	160 (42.9)	
Olive oil	409 (2.1)	54 (1.2)	22 (0.9)	327 (2.8)	5 (1.3)	
Margarine	6587 (34.5)	1567 (34.7)	1180 (47.6)	3701 (31.7)	116 (31.1)	
Low-fat margarine	4324 (22.6)	965 (21.4)	631 (25.4)	2665 (22.8)	50 (13.4)	
None	2067 (10.8)	564 (12.5)	237 (9.6)	1218 (10.4)	42 (11.3)	
Frequency of garlic, hot spices, ginger						<0.001
0 per week	2667 (13.8)	590 (12.9)	498 (19.7)	1511 (12.7)	68 (18.0)	
1–2 days per week	8013 (41.3)	2054 (45.0)	1191 (47.1)	4591 (38.7)	154 (40.7)	
3–4 days per week	5061 (26.1)	1182 (25.9)	541 (21.4)	3235 (27.3)	89 (23.5)	
>5 days per week	3634 (18.7)	742 (16.2)	297 (11.8)	2322 (21.3)	67 (17.7)	
Coffee servings						<0.001
0 cups per day	2057 (15.2)	622 (17.3)	366 (21.3)	996 (12.6)	66 (21.4)	
<2 cups per day	3442 (25.4)	864 (24.1)	456 (26.5)	2055 (26.0)	58 (18.1)	
2–4 cups per day	7468 (55.1)	1938 (54.0)	832 (48.3)	4507 (57.0)	171 (55.3)	
>4 cups per day	598 (4.4)	164 (4.6)	68 (3.9)	350 (4.4)	14 (4.5)	
Decaffeinated coffee servings						<0.001
0 cups per day	3628 (64.9)	1176 (75.6)	604 (68.6)	1715 (57.4)	124 (83.2)	
<2 cups per day	1172 (21.0)	233 (14.9)	169 (19.2)	744 (24.9)	20 (13.4)	
2–4 cups per day	754 (13.5)	145 (9.3)	99 (11.3)	504 (16.9)	5 (3.4)	
>4 cups per day	33 (0.6)	-	8 (0.9)	23 (0.8)	-	
Black tea servings						<0.001
0 cups per day	2815 (31.7)	970 (48.4)	440 (33.6)	1307 (24.5)	91 (44.4)	
<2 cups per day	2334 (26.3)	499 (24.9)	298 (22.7)	1482 (27.8)	51 (24.9)	
2–4 cups per day	3225 (36.3)	480 (24.0)	520 (39.7)	2157 (40.4)	59 (28.8)	
>4 cups per day	503 (5.7)	54 (2.7)	53 (4.0)	392 (7.3)	-	
Green tea servings						<0.001
0 cups per day	3248 (49.8)	1045 (57.4)	551 (59.2)	1546 (43.3)	98 (55.1)	
<2 cups per day	2136 (32.8)	538 (29.5)	215 (23.1)	1322 (37.0)	52 (29.2)	
2–4 cups per day	1066 (16.2)	224 (12.3)	151 (16.2)	664 (18.6)	24 (14.6)	
>4 cups per day	70 (1.1)	14 (0.8)	14 (1.5)	39 (1.1)	-	
Other tea servings						<0.001
0 cups per day	3171 (47.3)	1046 (64.1)	491 (46.6)	1518 (39.6)	109 (66.9)	
<2 cups per day	1916 (28.6)	377 (23.1)	248 (23.5)	1254 (32.7)	39 (23.9)	
2–4 cups per day	1433 (21.4)	194 (11.9)	285 (27.0)	938 (34.5)	13 (8.0)	
>4 cups per day	179 (2.7)	14 (0.9)	30 (2.8)	133 (3.5)	-	
Snack food frequency						<0.001
Never	2573 (13.6)	527 (11.5)	308 (12.2)	1673 (14.7)	57 (15.1)	
1–2 times per week	8411 (44.4)	2056 (45.0)	1090 (43.1)	5089 (44.6)	157 (41.5)	
3–4 times per week	4963 (26.2)	1232 (27.0)	695 (27.5)	2911 (25.5)	106 (28.0)	
>5 times per week	2990 (15.8)	756 (16.5)	434 (17.2)	1736 (15.2)	58 (15.3)	
Dessert frequency						0.037
Never	1942 (10.0)	477 (10.4)	223 (8.8)	1194 (10.1)	43 (11.4)	
1–2 times per week	6485 (33.5)	1575 (34.5)	791 (31.3)	3966 (33.5)	131 (34.7)	
3–4 times per week	5000 (25.8)	1177 (25.8)	687 (27.2)	3024 (25.5)	98 (25.9)	
>5 times per week	5953 (30.7)	1339 (29.3)	827 (32.7)	3669 (31.0)	106 (28.0)	
Artificial sweetener use						<0.001
Never	15,816 (86.3)	3616 (85.5)	1860 (78.1)	10,027 (88.2)	313 (90.5)	
¼ of the time	393 (2.1)	97 (2.3)	60 (2.5)	233 (2.0)	-	
½ of the time	252 (1.4)	58 (1.4)	52 (2.2)	140 (1.2)	-	
¾ of the time	166 (0.9)	48 (1.1)	24 (1.0)	92 (0.8)	-	
Always	1701 (9.3)	408 (9.7)	385 (16.2)	882 (7.8)	26 (7.5)	
Regular soft drink frequency						<0.001
Never	6596 (37.1)	1387 (33.5)	726 (33.4)	4351 (39.2)	110 (33.4)	
1–3 times per month	7342 (41.2)	1833 (44.2)	814 (37.5)	4537 (40.8)	145 (44.1)	
1–4 times per week	2620 (14.7)	608 (14.7)	349 (16.1)	1612 (14.5)	45 (13.7)	
5–7 times per week	613 (3.4)	162 (3.9)	140 (6.4)	292 (2.6)	17 (5.2)	
>1 per day	629 (3.5)	153 (3.7)	143(6.6)	319 (2.9)	12 (3.6)	
Diet soft drink frequency						<0.001
Never	7778 (48.8)	1795 (50.5)	739 (39.4)	5111 (50.0)	133 (45.7)	
1–3 times per month	4762 (29.8)	1046 (29.4)	575 (30.7)	3036 (29.7)	94 (32.3)	
1–4 times per week	1898 (11.9)	357 (10.0)	236 (12.6)	1266 (12.4)	34 (11.7)	
5–7 times per week	681 (4.3)	185 (5.2)	142 (7.6)	340 (3.3)	13 (4.5)	
>1 per day	837 (5.2)	173 (4.9)	183 (9.8)	461 (4.5)	17 (5.8)	
Amount of regular or diet soft drink						<0.001
<12 ounces (<1 can/bottle)	7531 (58.8)	1754 (55.5)	1062 (59.2)	4544 (60.2)	151 (55 9)	
12–16 ounces (1 can/bottle)	4975 (38.9)	1328 (42.0)	689 (38.4)	2834 (37.6)	117 (43.3)	
>16 ounces (>1 can/bottle)	295 (2.3)	81 (2.6)	44 (2.5)	166 (2.2)	-	
Soy or fish sauce frequency						<0.001
Never	8104 (42.1)	1737 (38.1)	1153 (46.1)	5019 (42.6)	170 (45.1)	
Rarely	7360 (38.2)	1847 (40.5)	851 (34.0)	4496 (38.2)	146 (38.7)	
Sometimes	3650 (18.9)	936 (20.5)	473 (18.9)	2174 (18.5)	60 (15.9)	
Always	150 (0.8)	37 (0.8)	25 (1.0)	87 (0.7)	-	
Salt seasoning frequency						<0.001
Never	6996 (36.3)	1621 (35.5)	1107 (44.1)	4098 (34.7)	149 (39.5)	
Rarely	5934 (30.8)	1325 (29.0)	800 (31.9)	3684 (31.2)	111 (29.4)	
Sometimes	3685 (19.1)	908 (19.9)	387 (15.4)	2329 (19.7)	55 (14.6)	
Always	2682 (13.9)	708 (15.5)	214 (8.4)	1687 (14.3)	62 (16.4)	
Fast food restaurant frequency						<0.001
Never	1372 (7.7)	169 (4.7)	80 (3.5)	1068 (9.7)	26 (7.6)	
≤1 per month	8306 (46.7)	1799 (43.5)	981 (43.1)	5346 (48.7)	157 (45.8)	
2–4 times per month	7365 (41.4)	1913 (46.3)	1101 (48.3)	4184 (38.1)	144 (42.0)	
2–4 times per week	573 (3.2)	169 (4.1)	93 (4.1)	296 (2.7)	13 (3.8)	
>5 times per week	171 (1.0)	54 (1.3)	23 (1.0)	91 (0.8)	-	
Fat/oil most often used						<0.001
None	134 (0.7)	23 (0.5)	24 (0.9)	82 (0.7)	-	
Margarine	815 (4.2)	249 (5.4)	78 (3.1)	468 (4.0)	19 (5.0)	
Butter	210 (1.1)	58 (1.3)	9 (0.4)	133 (1.1)	9 (2.4)	
Other	803 (4.1)	170 (3.7)	131 (5.2)	489 (4.1)	10 (2.6)	
Olive oil	4429 (22.8)	1106 (24.2)	621 (24.6)	2607 (22.0)	78 (20.6)	
Canola	1341 (6.9)	245 (5.4)	264 (10.4)	808 (608)	21 (5.6)	
Combination of above	11,630 (60.0)	2720 (59.5)	1402 (55.4)	7244 (61.2)	238 (63.0)	

- Data suppressed due to small cell counts.

**Table 4:** Association between BMI and frequency/servings of dietary variables.

Variable	*R*	Variable	*R*
Frequency of garlic, hot spices, ginger	−0.048 *^,†^	Servings of green tea	−0.029 *^,†,§^
Frequency of ≥5 fruit and vegetables	−0.081 *^,†^	Servings of other tea	0.024 ^†,§^
Servings of green vegetables	−0.032 *^,†^	Frequency of snack food	0.091 *^,†,§^
Servings of whole grains	−0.059 *^,†,§^	Frequency of desserts	0.005 ^†,§^
Servings of refined grains	0.075 *^,†^	Frequency of sweetener use	0.101 *^,†,§^
Servings of milk products	0.013 ^§^	Frequency of fruit juice	−0.042 *^,§^
Servings of eggs	−0.012 ^§^	Frequency of regular soft drink	0.111 *^,†^
Servings of meat/poultry	0.106 *^,†,§^	Frequency of diet soft drink	0.170 *^,†,§^
Servings of fish	0.018 *^,†,§^	Frequency of soy sauce	−0.005 ^§^
Servings of tofu	−0.050 *^,†^	Frequency of seasoning with salt	0.003 ^†^
Servings of beans	−0.011	Frequency of fast food restaurant	0.162 *^,†,§^
Servings of nuts/seeds	−0.058 *^,†^	Servings of vegetables	−0.069 *^,†^
Servings of coffee	0.032 *^,†^	Servings of fruit	−0.051 *^,†^
Servings of decaffeinated coffee	0.012 ^†,§^	Servings of fruit/vegetable juice	−0.015 *^,§^
Servings of black tea	0.008 ^†,§^	HEI	−0.073 *^,†,§^

Results are expressed as Spearman’s correlation coefficients for associations with BMI. * Indicates statistically significant association with BMI (*p* < 0.05). ^†^ Indicates statistically significant association found with waist circumference (*p* < 0.05), coefficients not shown. ^§^ Indicates statistically significant association found with percentage body fat (*p* < 0.05), coefficients not shown.

**Table 5:** Regression models for servings of dietary components as predictors of obesity ^†^.

Variable	Prevalence Ratio (95%CIs)	Variable	Prevalence Ratio (95%CIs)
Servings of green vegetables		Servings of nuts/seeds	
Unadjusted	0.95 (0.92–0.98)	Unadjusted	0.90 (0.87–0.93)
Model 1	0.95 (0.92–0.98)	Model 1	0.90 (0.87–0.93)
Model 2	0.97 (0.95–1.04)	Model 2	0.92 (0.89–0.95)
Model 3	0.98 (0.94–1.00)	Model 3	0.92 (0.89–0.95)
Servings of whole grains		Servings of coffee	
Unadjusted	0.95 (0.94–0.97)	Unadjusted	1.02 (1.00–1.04)
Model 1	0.95 (0.94–0.97)	Model 1	1.02 (1.00–1.03)
Model 2	0.97 (0.95–0.99)	Model 2	1.02 (1.00–1.04)
Model 3	0.97 (0.96–0.99)	Model 3	1.02 (1.00–1.04)
Servings of refined grains		Servings of decaffeinated coffee	
Unadjusted	1.09 (1.07–1.11)	Unadjusted	1.02 (0.98–1.06)
Model 1	1.08 (1.06–1.10)	Model 1	1.03 (0.99–1.08)
Model 2	1.07 (1.05–1.09)	Model 2	1.03 (0.99–1.08)
Model 3	1.06 (1.05–1.07)	Model 3	1.04 (1.00–1.09)
Servings of milk products		Servings of black tea	
Unadjusted	0.99 (0.97–1.01)	Unadjusted	1.00 (0.98–1.02)
Model 1	0.99 (0.97–1.01)	Model 1	1.00 (0.98–1.03)
Model 2	0.99 (0.97–1.01)	Model 2	1.00 (0.98–1.02)
Model 3	0.99 (0.97–1.01)	Model 3	1.01 (0.99–1.03)
Servings of eggs		Servings of green tea	
Unadjusted	1.01 (1.00–1.02)	Unadjusted	0.97 (0.94–1.01)
Model 1	1.01 (1.00–1.02)	Model 1	0.98 (0.94–1.01)
Model 2	1.01 (1.00–1.02)	Model 2	0.99 (0.96–1.03)
Model 3	1.02 (1.01–1.03)	Model 3	1.00 (0.96–1.04)
Servings of meat/poultry		Servings of vegetables	
Unadjusted	1.06 (1.05–1.07)	Unadjusted	0.95 (0.93–0.96)
Model 1	1.06 (1.05–1.07)	Model 1	0.95 (0.93–0.96)
Model 2	1.06 (1.06–1.07)	Model 2	0.97 (0.96–0.99)
Model 3	1.05 (1.04–1.06)	Model 3	0.97 (0.96–0.99)
Servings of fish		Servings of fruit	
Unadjusted	1.03 (0.99–1.06)	Unadjusted	0.95 (0.94–0.97)
Model 1	1.03 (1.00–1.07)	Model 1	0.95 (0.94–0.97)
Model 2	1.04 (1.01–1.07)	Model 2	0.97 (0.95–0.99)
Model 3	1.04 (1.01–1.07)	Model 3	0.97 (0.95–0.99)
Servings of tofu		Servings of fruit juice	
Unadjusted	0.82 (0.73–0.93)	Unadjusted	0.99 (0.97–1.01)
Model 1	0.83 (0.73–0.94)	Model 1	0.99 (0.96–1.01)
Model 2	0.84 (0.74–0.96)	Model 2	1.00 (0.97–1.02)
Model 3	0.84 (0.73–0.95)	Model 3	1.00 (0.97–1.02)
Servings of beans		HEI	
Unadjusted	0.98 (0.94–1.02)	Unadjusted	0.99 (0.99–0.99)
Model 1	0.98 (0.94–1.02)	Model 1	0.99 (0.99–0.99)
Model 2	1.00 (0.96–1.05)	Model 2	1.00 (0.99–1.00)
Model 3	1.00 (0.96–1.04)	Model 3	1.00 (0.99–1.00)

Model 1, adjusted for sex and age; Model 2, adjusted for age, sex, education, smoking, alcohol use and physical activity; Model 3, adjusted for age, sex, education, smoking, alcohol use, physical activity and province. ^†^ Obesity is defined as a BMI ≥30.0 kg/m^2^.
