# Correction: DeClercq, V.; et al. Association between Diet Quality and Adiposity in the Atlantic PATH Cohort. *Nutrients* 2017, *9*, 1155

**DOI:** 10.3390/nu10050540

**Published:** 2018-04-26

**Authors:** Vanessa DeClercq, Yunsong Cui, Cynthia Forbes, Scott A. Grandy, Melanie Keats, Louise Parker, Ellen Sweeney, Zhijie Michael Yu, Trevor J. B. Dummer

**Affiliations:** 1 Population Cancer Research Program, Department of Pediatrics, Dalhousie University, Halifax, NS B3H 4R2, Canada; yunsong.cui@dal.ca (Y.C.); cynthia.forbes@dal.ca (C.F.); louise.parker@dal.ca (L.P.); ellen.sweeney@dal.ca (E.S.); zhijie.m.yu@gmail.com (Z.M.Y.); 2 School of Health and Human Performance, Dalhousie University, Halifax, NS B3H 1T8, Canada; scott.grandy@dal.ca (S.A.G.); melanie.keats@dal.ca (M.K.); 3 Centre of Excellence in Cancer Prevention, School of Population and Public Health, University of British Columbia, Vancouver, BC V6T 1Z3, Canada; tdummer@mail.ubc.ca

The authors request the following corrections to their paper [1].

In the results section, “reported consuming <2 servings of whole grains and 54% reported consuming <2 servings of refined grains” was replaced with “reported consuming 1–2 servings of whole grains and 54% reported consuming 1–2 servings of refined grains”.

In Tables 2 and 3, all “>5” were replaced with “≥5”. 

In Table 2, the serving categories for green vegetables, whole grains and refined grains were amended to “0 per day”, “1–2 per day”, “3–4 per day”, and “≥5 per day”.

The authors apologize for this oversight and any inconvenience caused to the readers by these corrections, stating it does not affect the scientific results.
